# Identification of novel long non-coding RNAs deregulated in hepatocellular carcinoma using RNA-sequencing

**DOI:** 10.18632/oncotarget.7364

**Published:** 2016-02-13

**Authors:** Davide Degli Esposti, Hector Hernandez-Vargas, Catherine Voegele, Nora Fernandez-Jimenez, Nathalie Forey, Brigitte Bancel, Florence Le Calvez-Kelm, James McKay, Philippe Merle, Zdenko Herceg

**Affiliations:** ^1^ Epigenetics Group, International Agency for Research on Cancer (IARC), Lyon, France; ^2^ Genetic Cancer Susceptibility Group, International Agency for Research on Cancer (IARC), Lyon, France; ^3^ Centre de Recherche en Cancérologie de Lyon, UMR INSERM 1052, CNRS 5286, Lyon Cedex, France; ^4^ Hospices Civils de Lyon, Service d'Anatomopathologie, Groupement Hospitalier Lyon Nord, Lyon, France; ^5^ Hospices Civils de Lyon, Service d'Hépatologie et de Gastroentérologie, Groupement Hospitalier Lyon Nord, Lyon, France

**Keywords:** hepatocellular carcinoma, long non-coding RNA, gene networks, enhancer-associated RNAs, RNA-sequencing

## Abstract

Functional characterization of long non-coding RNAs (lncRNAs) and their pathological relevance is still a challenging task. Abnormal expression of a few long non-coding RNAs have been found associated with hepatocellular carcinoma, with potential implications to both improve our understanding of molecular mechanism of liver carcinogenesis and to discover biomarkers for early diagnosis or therapy. However, the understanding of the global role of lncRNAs during HCC development is still in its infancy. In this study, we produced RNA-Seq data from 23 liver tissues (controls, cirrhotic and HCCs) and applied statistical and gene network analysis approaches to identify and characterize expressed lncRNAs. We detected 5,525 lncRNAs across different tissue types and identified 57 differentially expressed lncRNAs in HCC compared with adjacent non-tumour tissues using stringent criteria (FDR<0.05, Fold Change>2). Using weighted gene co-expression network analysis (WGCNA), we found that differentially expressed lncRNAs are co-expressed with genes involved in cell cycle regulation, TGF-β signalling and liver metabolism. Furthermore, we found that more than 20% of differentially expressed lncRNAs are associated to actively transcribed enhancers and that the co-expression patterns with their closest genes change dramatically during HCC development. Our study provides the most comprehensive compendium of lncRNAs expressed in HCC, as well as in control or cirrhotic livers. Our results identified both known oncogenic lncRNAs (such as H19 and CRNDE) and novel lncRNAs involved in cell cycle deregulation and liver metabolism deficits occurring during HCC development.

## INTRODUCTION

Liver cancer is the second most common cause of cancer-related mortality worldwide, and is estimated to be responsible for nearly 746,000 deaths in 2012 (9.1% of the total). Liver cancer is largely a problem of the less developed regions where 83% of the estimated 782,000 new cancer cases worldwide occurred in 2012 [1]. Hepatocellular carcinoma (HCC) is the most common type of liver cancer, accounting for about 80% of the tumours in this organ [2]. The most important etiological factors remain viral infections (typically involving HBV and HCV), aflatoxin exposure, and alcohol consumption [3]. However, obesity, metabolic syndrome, and diabetes have recently been recognized as risk factors for HCC and due to their increased incidence in both developed and developing countries, metabolic diseases will lead to more cases of HCC [4–6].

The molecular pathways involved in the development of HCC are diverse and no universal molecular feature has been found associated with all hepatic tumours. Recent genome-wide sequencing studies have confirmed the occurrence of gene mutations previously identified, particularly affecting the p53, the retinoblastoma (Rb), the TGF-β and the Wnt/β-catenin pathways. Moreover, these studies found new frequent mutations in various components of chromatin modifying complexes, particularly ARID1A or ARID2 [7–10]. Epigenetic alterations may also contribute to HCC molecular and clinicopathological heterogeneity. Notably, our group identified DNA methylation signatures able to distinguish HCC from paired-matched non-tumour surrounding tissues and HCC according to the different aetiologies [11–12]. Recently, genome-wide aberrant DNA methylation patterns in HCC tumours that are predominantly HCV-related were reported [13]. A total of 2,568 significant CpG sites were located within 684 differentially methylated regions which covered 589 genes, with some of them associated with HCV infection and/or cirrhosis, demonstrating the significance of aberrant DNA methylation in HCC tumourigenesis [13].

In the last decade, a further layer of the epigenetic control of gene transcription has emerged, namely long-non coding RNAs (lncRNAs). Interest in this field was stimulated by the finding that almost all of the mammalian genome is transcribed, while only about 2% of the genome sequences encodes proteins [14–15]. Long non-coding RNAs are generally defined as endogenous cellular RNAs longer than 200 nucleotides, expressed typically at lower levels than protein-coding mRNAs. Functionally, most of known or predicted lncRNAs have not been characterized, although for few of them mechanistic studies showed important roles mainly in mammal development and cell differentiation, while their deregulation has been detected in diseases, notably cancer [16–19].

Currently, few lncRNAs have been described to be deregulated in HCC and their aberrant expression was associated with tumourigenesis, metastatic disease, or prognostic/diagnostic [20]. In particular, the lncRNA H19, an imprinted gene in the IGF2 locus, was found to be upregulated in HBV associated HCC and induced by c-Myc and hypoxia [17, 21]. Moreover, H19 was shown to induce the expression of the multi-drug resistance gene MDR1 in liver cancer cells through regulation of its promoter methylation [22].

HULC is considered the first lncRNA specifically upregulated in HCC [23]. Recently, it was shown that HULC functions as an oncogene in hepatoma cells, acting mechanistically by promoting lipogenesis and disturbing the Clock circadian regulator (CLOCK)/brain and muscle arnt-like protein-1 (BMAL1) complex [24, 25]. The latter governs the regulation of circadian rhythm in hepatoma cells [25]. The lncRNA HOX transcript antisense RNA (HOTAIR) have also been found to be significantly overexpressed in HCC tissues [26]. Recently, a regulatory network between miR-218 and HOTAIR was shown, in which HOTAIR negatively regulated miR-218 expression in HCC through EZH2 targeting miR-218-2 promoter, resulting in the overexpression of the oncogene Bmi-1 [27]. However, only two genome-wide studies focussed specifically on lncRNAs in HCC and both of them were based on micro-array approaches. The findings showed deregulation of hundreds of lncRNAs. One study was based on the analysis of five cases of HBV-associated HCC and the second one on the analysis of 3 cases [28, 29].

In this study, we aimed to identify lncRNA expressed in HCC, cirrhotic and normal liver tissues using RNA-sequencing (RNA-Seq) and to infer their potential impact on gene transcription control and signalling pathways relevant to human hepatocarcinogenesis.

## RESULTS

### The lncRNAs landscape of HCC

**Figure 1:**
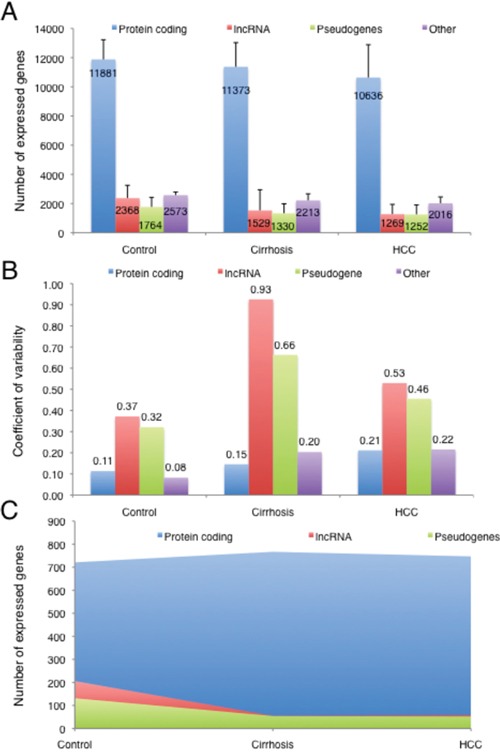
**A.** Number of genes expressed in control livers (3 cases), cirrhotic livers adjacent to HCCs and HCCs (10 cases) for each different gene category. **B.** Expression variability of each gene category in control livers, cirrhotic livers adjacent to HCCs and HCCs. **C.** Uniquely expressed genes for each category in control livers, cirrhotic livers adjacent to HCCs and HCCs.

Since a reference expression landscape for lncRNAs specific for HCC and non-tumour livers has not been described yet in the literature, we first analysed the sequencing data obtained in HCC (10 samples), tumour-matched cirrhotic livers (10 samples) and control livers (3 samples). It must be noted that control samples were not fully healthy livers, since they received pre-operative chemotherapy for liver metastases. Chemotherapy was associated with mild liver toxicity that was histologically recognized by steatosis or fibrosis. Therefore, although the treatment of these cases was stopped one month before surgery, we cannot exclude some residual effects of the treatment on gene expression and potentially under- or overestimation of lncRNAs expression in these tissues. A total of 857.7 million (M) of reads were mapped, with a mean of 37.3 M of reads per sample (range 11.2-72.1 M), of which 6.2% were mapped to annotated long non-coding RNAs (Table 1). In order to depict a tissue specific landscape of gene expression, we kept only genes expressed above a threshold of 1 FPKM in at least half of the samples for each tissue type, a threshold typically used to avoid potential confounding effects from transcriptional noise [15]. Then, we analysed the number of expressed genes in each sample for each tissue type, considering separately 3 gene categories, namely protein coding genes, long non-coding genes (lncRNAs), and pseudogenes, as they are defined in Gencode annotation (release 17). The genes not included in these categories were classified as “other”. On average, the total number of genes expressed in control livers, cirrhotic or HCC tissues was 18,586±3,006; 16,445±4,131 and 15,172±3551, respectively. While a decreasing trend in the number of total expressed genes seems to appear from control to cirrhotic and HCC tissues, this difference was not significant. No significant differences were observed when comparing different gene categories for each tissue type either (Figure 1A). However, the number of expressed genes for lncRNAs showed a higher variability (measured as coefficient of variation) than protein-coding genes or pseudogenes in each tissue type, with the highest level of variability observed in cirrhotic livers (Figure 1B).

**Table 1:** RNA-Sequencing general data after alignment and mapping

Sample	Total mapped reads^a^(Millions)	Total mapped reads^b^(Millions)	% of lncRNAs reads
112N	30.2	1.5	4.9
112T	27.7	1.8	6.4
122N	11.2	0.7	5.9
122T	16.1	1.0	6.2
127N	21.8	1.0	4.7
127T	21.9	1.2	5.3
1N	33.6	2.2	6.4
1T	25.6	1.6	6.3
2N	75.3	4.2	5.5
2T	46.9	3.0	6.5
8N	46.4	2.8	6.0
8T	72.1	4.1	5.7
20N	61.6	3.5	5.7
20T	52.7	4.2	8.0
13N	49.0	3.4	7.0
13T	55.6	4.0	7.2
14N	42.0	2.2	5.2
14T	37.2	3.0	8.1
15N	29.2	1.5	5.1
15T	27.7	1.8	6.5
C1	24.9	1.4	5.8
C5	29.8	1.7	5.8
C8	19.4	1.2	6.2
TOTAL	857.7	53.0	6.2
Average	37.3	2.3	6.2

a Mapped and annotated using Genecode V17 annotation

b Number of reads corresponding to lncRNAs annotated in Genecode V17 annotation

We further analysed the gene expression landscape taking into consideration the genes that are uniquely expressed in each tissue type, in order to assess the histopathological specificity of each gene category. Furthermore, to identify the uniquely expressed genes, we compared the list of genes expressed over 1 FPKM in control livers, cirrhotic or HCC tissues and we kept only those that were commonly expressed across all samples of each tissue type. Then, we identified those that were uniquely expressed in each tissue type. Interestingly, we observed a statistically significant decrease (p<0.001, χ^2^ test) in the number of uniquely lncRNAs and pseudogenes in cirrhotic and HCC tissues compared with control livers, while no differences were observed for protein-coding genes (Figure 1C, Table 2).

**Table 2:** Uniquely expressed genes in control, cirrhotic livers or hepatocellular carcinomas (HCC)

Histology	Uniquely expressed genes						
	Protein coding		Long non coding RNAs		Pseudogenes		TOTAL
	N°	%	N°	%	N°	%	
Control livers	721	3.9	206	1.1	131	0.7	18,586
Cirrhotic livers	767	4.7	55^***^	0.3	54^***^	0.3	16,445
HCC	747	4.9	58^***^	0.4	52^***^	0.3	15,172

χ^2^ test:

*** p-value<0.001 compared with controls

Overall, these results suggest that lncRNAs are an important element in the transcriptional landscape of hepatic gene expression and the loss of expression of lncRNAs may be an early event during HCC development.

### LncRNAs are differentially expressed in HCC

In order to identify lncRNAs differentially expressed in HCC tissues compared with paired-matched adjacent cirrhotic tissues, we performed a differential expression analysis using the edgeR package, which is considered as a robust method to analyse RNA-Seq data [30, 31]. After filtering, a total number of 29,827 genes across all the samples were kept for the analysis, with 18,377 protein-coding genes, 5,525 lncRNAs and 3,939 pseudogenes.

**Figure 2:**
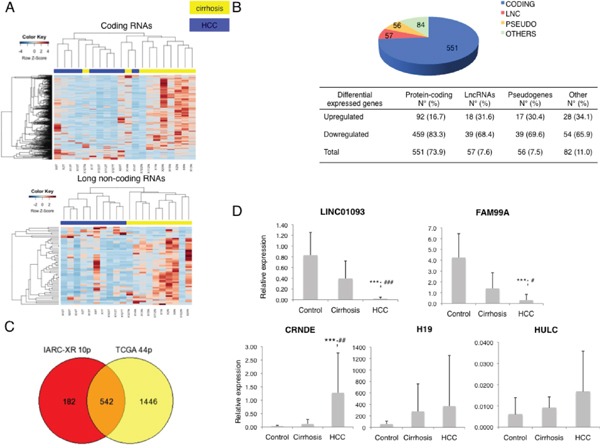
**A.** Heatmaps showing unsupervised clustering of HCC and adjacent cirrhotic tissues based on differentially expressed coding genes (up) and long non-coding genes (down). **B.** Distribution of differentially expressed genes by gene category. **C.** Overlap between differentially expressed genes in IARC-Croix Rousse cohort and TCGA paired matched cases, annotated with RefSeqGene. **D.** RT-qPCR results on 5 different lncRNAs in 20 cases (HCC and cirrhotic adjacent tissues) and 10 control livers.

The results showed 746 differentially expressed genes with a FDR<0.05 and a FC>|2|. One hundred and forty-five genes were upregulated and 601 genes were downregulated in HCC compared with cirrhotic tissues. Protein-coding genes accounted for 73.9% of the deregulated genes, with 92 upregulated and 459 downregulated in HCC (Figure 2A). LncRNAs and pseudogenes accounted together for 15% of the deregulated genes, with 18 upregulated and 39 downregulated genes for lncRNA (Figure 2A, Table 3 and 17 upregulated and 39 downregulated genes for pseudogenes (Figure 2B, Supplementary Document 1). Importantly, we observed a better clustering using only differentially expressed lncRNAs than when using differentially expressed protein-coding RNAs or all differentially expressed genes. Interestingly, 4 upregulated and 9 downderegulated lncRNAs in HCC (22.8% of all deregulated lncRNAs) have been recently described as lncRNAs associated with predicted enhancer activity [32]. A pathway analysis of upregulated protein-coding genes using Enrichr [33] showed an enrichment in genes mainly involved in cell cycle (p-value=0.0008) and angiogenesis (Supplementary Document 2). Concerning the downregulated protein-coding genes, the pathway analysis showed an enrichment in genes involved in metabolic pathways and liver functions, particularly the cytochrome P450 family and the complement system (Supplementary Document 3).

**Table 3:** Differentially expressed long non-coding RNAs in HCC compared with adjacent cirrhotic tissues

Genes	Gene symbol	Expression levels in cirrhosis (FPKM)	Expression levels in HCC (FPKM)	FDR^(a)^
ENSG00000228651.1	RP11-556E13.1	0.32	6.21	0.00003
ENSG00000253819.1	LINC01151	0.65	2.77	0.04545
ENSG00000257579.1	-	0.26	0.90	0.02398
ENSG00000250696.1	RP11-704M14.1	1.19	3.16	0.00097
ENSG00000233396.1	-	0.21	0.72	0.00567
ENSG00000228709.1	-	0.58	1.79	0.01721
ENSG00000267583.1	-	0.33	0.78	0.00640
ENSG00000230325.1	-	0.54	1.32	0.03730
ENSG00000228918.1	LINC01344	0.40	0.85	0.00056
ENSG00000261716.1	-	0.79	1.73	0.00150
ENSG00000243533.1	-	2.17	4.23	0.01998
ENSG00000230850.3	-	0.10	0.24	0.01509
ENSG00000232995.2	RGS5	1.09	2.39	0.00016
ENSG00000245694.4	CRNDE	0.74	1.54	0.00577
ENSG00000259347.1	-	2.48	5.45	0.02980
ENSG00000237036.3	-	0.47	1.04	0.00976
ENSG00000243628.2	-	0.56	1.22	0.01902
ENSG00000270933.1	-	0.99	1.75	0.01110
ENSG00000230333.2	-	1.26	0.59	0.01619
ENSG00000225431.1	LOC101928233	0.77	0.38	0.02902
ENSG00000233421.2	-	8.44	2.80	0.04678
ENSG00000233695.1	-	1.55	0.72	0.00446
ENSG00000258867.1	LINC01146	5.71	3.52	0.01950
ENSG00000249201.2	CTD-3080P12.3	1.78	0.77	0.00345
ENSG00000237517.4	DGCR5	1.37	0.57	0.01325
ENSG00000228826.1	-	2.56	1.05	0.01722
ENSG00000236341.1	-	2.17	0.84	0.00005
ENSG00000233554.1	-	1.92	0.76	0.00266
ENSG00000233392.1	LOC200772	0.90	0.34	0.00177
ENSG00000232310.1	-	0.81	0.30	0.00008
ENSG00000254192.1	-	0.73	0.24	0.01924
ENSG00000229005.1	HNF4A-AS1	5.41	2.34	0.01208
ENSG00000249364.1	LOC101928858	2.23	0.79	0.03451
ENSG00000223956.1	-	3.32	1.41	0.00688
ENSG00000239685.1	-	30.93	12.76	0.00245
ENSG00000224652.1	LINC00885	1.04	0.36	0.00044
ENSG00000248740.1	LOC101929448	2.20	0.59	0.00004
ENSG00000230613.1	HM13-AS1	0.98	0.36	0.02403
ENSG00000261058.1	-	1.81	0.58	0.00428
ENSG00000253364.1	-	0.89	0.27	0.00038
ENSG00000229740.1	-	2.73	0.84	0.00516
ENSG00000257878.1	-	2.85	0.82	0.00000
ENSG00000250463.1	-	1.50	0.41	0.00005
ENSG00000264066.2	-	38.03	8.67	0.00219
ENSG00000221857.3	-	53.23	15.66	0.00007
ENSG00000255071.1	SAA2-SAA4	143.42	55.29	0.01047
ENSG00000049319.2	-	9.72	4.00	0.01193
ENSG00000205865.4	FAM99B	0.69	0.18	0.00370
ENSG00000258442.1	-	2.23	0.44	0.00000
ENSG00000237949.1	-	13.00	5.52	0.02010
ENSG00000243694.2	-	2.77	0.41	0.00000
ENSG00000262588.1	-	7.64	2.71	0.00021
ENSG00000240801.1	-	339.18	43.36	0.00727
ENSG00000249173.1	-	6.95	0.61	0.00000
ENSG00000130600.10	H19	222.04	123.02	0.00004
ENSG00000231486.3	-	147.04	52.01	0.00014
ENSG00000205866.2	FAM99A	5.34	0.91	0.00002

(a) False Discovery Rate. The FDRs reflect statistical significance from the differential analysis performed using read counts as expression units (see Materials and Methods section)

In order to take into account the effects of transcriptional noise at low levels of expression, we verified the level of expression of differentially expressed lncRNAs in each sample. All lncRNAs found upregulated in HCC were identified with at least 10 reads/sample and 10 had more than 50 reads/sample. Similarly, all lncRNAs found downregulated in HCC were identified with more than 20 reads/sample in the adjacent non tumour tissues, with 25 having more than 50 reads/sample.

In order to validate these results, we used the gene expression data available from The Cancer Genome Atlas for HCC. We analysed 44 cases for which HCC and paired-matched non tumour adjacent tissues were available. The annotated data available from TCGA have a large overlap with RefSeqGene annotation (20,031 genes out of 20,500 included in TCGA gene list, namely 97.7% of the genes). We thus annotated our sequencing data *ex-novo* using RefSeqGene annotation provided in LifeScope. After differential expression analysis using edgeR, we found 1988 differentially expressed genes in the TCGA dataset and 724 differentially expressed genes in our dataset (FDR<0.05 and FC>|2|). Indeed, 542 genes were in common (p-value<1.615e-298), corresponding to 75% of the genes we found differentially expressed (Figure 2C).

We further analysed the expression of a subset of differentially expressed lncRNAs in our cohort of 23 HCC cases and 10 controls. We performed RT-qPCR on 3 highly downregulated lncRNAs in HCC, namely *FAM99A*, *LINC01093*, and *H19*, and on one highly upregulated lncRNAs, namely *CRNDE*. We also performed RT-qPCR on *HULC*, a previously described lncRNAs upregulated in HCC that we did not find among the deregulated lncRNAs. We found that *LINC01093, FAM99A* and *CRNDE* were differentially expressed in HCC compared with cirrhotic tissues, confirming the data we obtained in the RNA-Seq experiment. Moreover, we found that *LINC01093* and *FAM99A* were already significantly downregulated in cirrhotic tissues compared with normal livers (Figure 2D). We were not able to validate the differential expression of *H19* (Figure 2D). Interestingly, H19 has been reported to be either upregulated or downregulated in HCC compared with non tumour liver, suggesting a high variability across different cohorts of patients [21, 34]. The absence of differential expression of *HULC* in our cohort was confirmed as no significant differences were observed in HCC compared to cirrhotic or normal livers.

In conclusion, our data suggest that changes in the expression of lncRNAs could play an important role in HCC development, as these may already occur at the cirrhotic stage and at a level of expression beyond what may be considered transcriptional noise.

### LncRNAs associated with transcribed enhancers show characteristic patterns of co-expression during HCC development

Gene co-expression analysis is based on the assumption that genes that have similar expression patterns across a set of samples may have a functional relationship. This approach may give different and complementary information to differential expression analysis. In our differential expression analysis we found 13 lncRNAs associated with transcribed enhancers (eRNAs). Thus, in order to investigate the effects of their altered expression on neighbouring genes, we systematically analysed their co-expression patters with their closest 96 genes (48 genes upstream and 48 genes downstream). We calculated Pearson's correlation coefficients for each gene pair and created correlation matrixes for each genomic region using gene expression data from our RNA-Seq data.

**Figure 3:**
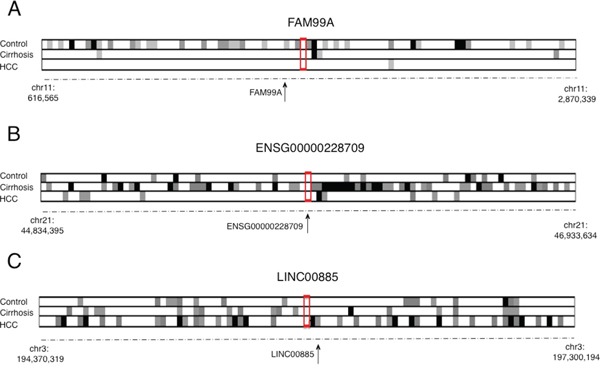
Co-expression analysis of 3 different enhancer-associated lncRNA (eRNAs), representing the 3 main trends observed in the co-expression patterns of differentially expressed eRNAs Each small square represents the P value for the correlation of the expression level in a specific gene pair. White, light gray, dark gray and black indicate Pearson's correlation P values of >0.05, <0.05, <0.01 and <0.001, respectively. **A.** Co-expression analysis for FAM99A. Loss of co-expression is observed in cirrhotic and HCC samples compared with control livers (χ^2^ test, p-value<0.05). **B.** Co-expression analysis for ENSG00000228709) Gain of co-expression in cirrhotic tissues compared with control livers or HCCs (χ^2^ test, p-value<0.05). **C.** Co-expression analysis for LINC00885. Gain of co-expression is observed in HCC compared with cirrhotic or control livers (χ^2^ test, p-value<0.05).

Interestingly, we observed three different trends in the co-expression pattern of the 13 different eRNAs analysed (Figure 3). In particular, 4 genes showed a significant (p-value<0.05) loss of co-expressed genes in both cirrhotic livers and HCC compared with control livers (*FAM99A, ENSG00000223956, ENSG00000237949, ENSG00000225431*). Of note, the loss of co-expression is associated with the loss of expression in HCC compared with cirrhotic livers for these 4 genes, suggesting the loss of co-expression could occur earlier than the loss of expression. Six additional eRNAs, 2 upregulated (*LINC01344, ENSG00000228709*) and 4 downregulated (*FAM99B*, *ENSG00000237517, ENSG00000253364, ENSG00000249201*), showed the most different patterns of co-expression in cirrhotic tissues (p-value<0.05). Four eRNAs (*LINC01344, ENSG00000228709, FAM99B, ENSG00000237517)* and two eRNAs *(ENSG00000253364, ENSG00000249201*) showed the highest number and the lowest number of co-expressed genes in the cirrhotic tissues, respectively. A single eRNA (LINC00885) among those analysed showed a higher number of co-expressed genes in HCC tissues compared with control or cirrhotic livers (p-value<0.05).

In order to verify if the co-expression patterns could be due to copy number alterations (CNA) of the genes in the selected regions, we used the data on the frequency of CNAs for 9 lncRNAs for which data were reported in cBioportal (http://www.cbioportal.org). Globally, CNAs were reported for 12 cases out of 377 HCC samples (from TCGA dataset) with *LINC00885* accounting for half of the cases (6 cases with amplification) (Supplementary Figure 1). Notably, we observed a downregulation of *LINC00885* in HCC compared with adjacent cirrhotic tissues. These data suggest that the observed co-expression patterns are not likely to be driven by subjacent genetic alterations, but they may result from altered transcription or epigenetic programmes in these genomic regions.

Overall, these results showed significant alterations of the co-expression patterns in 11 differentially expressed eRNAs in the different tissue types, suggesting specific transcriptional interactions between these enhancer-associated lncRNAs and their closest protein-coding genes during HCC development.

### Genome-wide co-expression network analysis identifies new long non-coding RNAs potentially involved in pathways related to HCC development

In order to infer the biological functions of differentially expressed lncRNAs and to identify gene clusters in which their expression is correlated with protein-coding RNAs, we used weighted gene co-expression network analysis (WGCNA, see Methods Section). Expression values from all sequenced samples were used in the analysis. In particular, we show the results obtained after analysing the 746 genes previously found differentially expressed in HCC compared with adjacent cirrhotic tissues.

**Figure 4:**
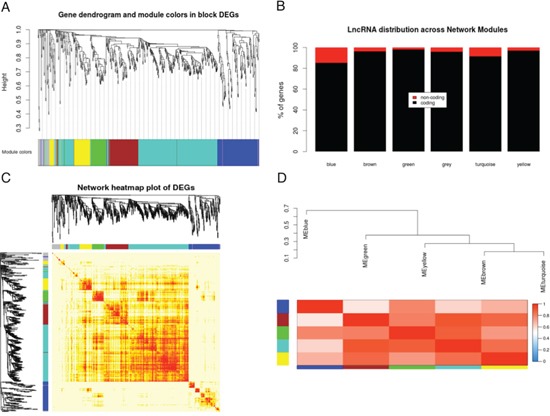
Gene co-expression network analysis of differentially expressed genes **A.** Gene clustering and module identification. Each co-expression cluster has a different colour. **B.** LncRNAs distribution in the different modules. **C.** Co-expression matrix showing relations across the different modules. **D.** Eigengene heatmap showing correlation among the different eigengene vectors of each module.

A step-by-step network construction and module detection method was used after choosing a selected power (power = 12) determined through a soft threshold approach and setting to 30 the minimum number of genes per module. Adjacent modules with a minimum cut Height of 0.15 were merged. Using these parameters, we clustered highly co-expressed genes into 5 co-expression modules (Figure 4A, Supplementary Document 4).

The module size ranged from 51 to 325 genes. A group of 48 non co-expressed genes was also identified and was labelled as “grey” following the standard output colour proposed by the authors. The percentage of lncRNAs in each module ranged from 1.9% (green module, 1 lncRNA) to 14.7% (blue module, 20 lncRNAs) (Figure 4B). The turquoise module had both the highest number of genes and of lncRNAs (325 genes of which 28 lncRNAs). Interestingly, genes in the blue module showed a lower correlation with most of the genes in the other modules, except for a few genes in the yellow and the green modules (Figure 4C). Its distance from the other gene modules was confirmed by eigengene network analysis (Figure 4D). Moreover, the blue module was characterized for harbouring more than 85% of the upregulated genes in HCC. In particular, all 18 upregulated lncRNAs and 15 out of 17 upregulated pseudogenes clustered in this module as well as 86 protein-coding genes out of 92 upregulated protein coding genes.

Pathway analyses and transcription factor enrichment analyses were performed on each single module (Supplementary Document 5). Our results showed that most of the genes in the blue module are involved in cell cycle, with a significant enrichment for genes that are under the transcriptional control of FOXM1. The remaining co-expression modules harboured only downregulated genes in HCC. The turquoise module was the second most enriched module in lncRNAs after the blue one. Genes in the turquoise module are mostly involved in metabolic pathways (the cytochrome P450 family, the complement system and retinol metabolism). Concerning the other three modules, both the brown and yellow modules were enriched in genes involved in inflammatory response, mainly driven by TGF-β signalling pathway and transcription factors of the SMAD and STAT families. Finally, the green module was enriched in genes involved in PPAR signalling pathway.

Interestingly, when all expressed genes from all tissues were included in the WGCNA analysis, similar results were found, in particular most of the upregulated lncRNAs (12 out of 18) clustered in the same module (data not shown), suggesting that the co-expression patterns were confirmed and likely to be functionally relevant.

Overall, these results suggest that most of the deregulated lncRNAs in HCC may be actively involved in cell cycle control and liver specific metabolic functions.

## DISCUSSION

The discovery that most of the human genome is transcribed while only 2% of it codes for proteins stimulated the interest in the field of non-coding RNAs [14, 35]. In particular, lncRNA transcripts have emerged as an important layer in the genetic regulatory code [35, 36]. However, while the number of annotated lncRNAs is growing, the characterization of their putative functions is still in its infancy. A number of lncRNAs have been found differentially expressed in cancer cells or tissues compared with their normal counterpart, suggesting a role in cancer development and progression [20, 37]. In this study, we used deep sequencing technology firstly to identify the expression profiles of lncRNAs in HCC, cirrhotic or control livers and then we applied differential expression and co-expression analyses to infer the potential biological relevance of lncRNAs during human HCC development. In agreement with previous studies [38, 39], we observed a higher variability in the number of expressed lncRNAs than protein coding genes or pseudogenes in each tissue type, including in control livers. LncRNAs expression variability was increased in cirrhotic livers and HCC compared with control livers, with highest levels observed in the tumour-adjacent cirrhotic tissues. While a potential mechanism to explain this observation remains unknown, maximal epigenetic diversity has been previously described in normal cells with increased risk of transformation and cancer development [40]. Thus, it is possible that global expression of long non-coding RNA, such as DNA methylation, is particularly sensitive to the carcinogenic process. Interestingly, it has been shown that genes with highly variable expression are associated with human disease and involved in development and extracellular response [41]. However, expression variability could also be due to transcriptional noise, in particular for most lncRNAs that are expressed at low levels, and potential functional implication only based on this feature should be interpreted with caution [42]. Finally, when uniquely expressed genes were taken into consideration in each tissue type, we observed a significant decrease in the number of expressed lncRNAs and pseudogenes in cirrhotic livers and HCC. One limitation of our study concerns the fact that control livers were not healthy livers, since they received pre-operative chemotherapy for liver metastases. However, mild lesions we found associated to chemotherapy (in particular steatosis) are also observed in healthy populations without liver disease symptoms. In particular, the prevalence of hepatic steatosis may range from 17-33% in the general population [43]. Moreover, our controls represent the first dataset of RNA-Seq data available from livers without chronic disease and could be of value for further studies on both healthy liver and chemotherapy-response gene expression.

Our differential and co-expression analyses excluded genes expressed at extremely low levels. Indeed, all lncRNAs found differentially expressed were identified with at least 10 reads/sample and more than 60% of them were identified with more than 50 reads/sample. Finally, we observed different co-expression patterns in different genomic contexts, suggesting that while a common pattern of co-expression could not be revealed for lncRNAs, this seems to be highly loci-dependent. Contrary to previous studies aimed at the analysis of lncRNAs in HCC [28, 29], which were based on microarray platforms, we performed RNA-seq, which is not biased by probe selection. Moreover, these two previous studies analysed primarily HBV-associated HCCs, while our series contained mostly patients with HCV- or alcohol-associated HCCs. Thus, differences in patient population, technology used and the limited number of samples may explain the different results in terms of number and type of lncRNAs found differentially expressed in the three studies. However, it is noteworthy that Yang et al. [28] studied the function of an upregulated lncRNAs (lncRNA-HEIH) in HCC, and showed its implication in cell cycle control. Indeed, our gene network analysis identified a cluster of 18 upregulated lncRNAs co-expressed with protein-coding genes involved in cell cycle regulation. Out of these, only CRNDE has been previously described as an upregulated gene in colorectal cancers and gliomas, responsive to PI3K/Akt/mTOR and MAPK pathways [44–46]. Thus, we identified novel co-expressed lncRNAs that may be potentially involved in cell cycle regulation during HCC development. Moreover, we observed that mostly downregulated lncRNAs were co-expressed with genes involved in liver metabolism. All environmental and lifestyle risk factors for HCC (HBV and HCV, alcohol consumption, aflatoxin B1 and metabolic syndrome) are known to induce chronic liver disease with a consequent loss of liver function. Interestingly, altered liver metabolism has also been associated with the modification of other epigenetic processes, such as DNA methylation, histone modification or microRNA expression [47]. It is worth noting that recent studies have described lncRNAs involved in lipid metabolisms [24, 48]. In particular, the lncRNA HULC, known to be overexpressed in a subset of HCCs, has been shown to deregulate lipid metabolism in HCC by activating the acyl-CoA synthetase subunit ACSL1, whose overexpression was sufficient to promote hepatoma cells proliferation [24]. Our data provide hints that many more lncRNAs may also be involved in the maintenance of liver metabolic functions. However, how these lncRNAs are functionally related to specific hepatic metabolic pathways remains to be elucidated.

In our study we also highlighted that more than 20% of the differentially expressed lncRNAs overlap with genomic loci described as enhancers [32]. In particular, we observed that 6 out of 11 eRNAs showed significant alterations in the tumour-adjacent cirrhotic tissues, with either gain or loss of co-expressed genes compared with control livers or paired matched HCCs. This result suggests that changes in eRNAs co-expression patterns could play a role in the early event of HCC development, even before a change in the expression levels. Moreover, changes in co-expression patterns were observed in genes located in genomic region spanning 1-2 Mb from the lncRNAs analysed, suggesting that these eRNAs may play a role as dynamic molecular mediators of transcriptional activation, as previously hypothesized [49].

In conclusion, our study provides a novel compendium of lncRNAs expressed in control livers, as well as cirrhotic livers and paired matched HCCs. Our analysis identified new lncRNAs differentially expressed during HCC development, with notably more than 20% associated with predicted enhancer activity. Altered expression of some of these enhancer-associated lncRNAs leads to dramatic changes of co-expression patterns in the different tissue type, suggesting that some lncRNAs may function as hub genes controlling the expression of the associated genes. Our gene network analyses identified 18 novel lncRNAs highly co-expressed with genes involved in cell cycle regulation, suggesting that lncRNAs may be effectively involved in cell cycle disruption during HCC development. Moreover, we also identified at least 28 novel lncRNAs whose expression is highly correlated with genes involved in liver metabolism, suggesting that many lncRNAs may also be involved in specific liver functions that are lost during chronic liver disease.

## MATERIALS AND METHODS

### Study population and experimental design

This study is based on a series of 33 patients who underwent hepatic resection either for hepatocellular carcinomas (HCC) (23 patients) or for liver metastases (10 patients) at the Croix Rousse Hospital, Lyon, France. Patient characteristics are shown in Table 4.

**Table 4:** Patients characteristics

Patient No.	Age	Gender	Status	Tissue^a^	Sequencing code	Primary aetiology	Secondary aetiology	EGS^c^
1	70	Male	Case	T+NT	1T, 1NT	NASH	-	3
2	75	Male	Case	T+NT	2T, 2NT	NASH	-	2
3	68	Female	Case	T+NT	N.A	NASH	-	3
4	70	Male	Case	T+NT	N.A	NASH	-	3
5	68	Male	Case	T+NT	N.A	NASH	-	2
6	70	Male	Case	T+NT	N.A	Alcohol	-	2
7	63	Male	Case	T+NT	N.A	Alcohol	Obesity	2
8	74	Male	Case	T+NT	8T, 8NT	Alcohol	-	2
9	60	Male	Case	T+NT	N.A	Alcohol	-	3
10	63	Male	Case	T+NT	N.A	Alcohol	-	4
11	55	Female	Case	T+NT	N.A	Alcohol	-	3
12	57	Male	Case	T+NT	N.A	Alcohol	-	3
13	74	Male	Case	T+NT	13T, 13NT	HCV	-	2
14	52	Male	Case	T+NT	14T, 14NT	HCV	NASH	3
15	44	Male	Case	T+NT	15T, 15NT	HCV	Alcohol	3
16	38	Male	Case	T+NT	N.A	HBV	-	3
17	67	Male	Case	T+NT	N.A	HBV	-	3
18	56	Male	Case	T+NT	N.A	HBV	-	3
19	21	Male	Case	T+NT	N.A	Cryptogenic	-	4
20	57	Female	Case	T+NT	20T, 20NT	Glycogenosis	-	3
21	77	Male	Case	T+NT	112T, 112NT	Alcohol	-	N.A
22	67	Male	Case	T+NT	122T, 122NT	Alcohol	-	N.A
23	79	Female	Case	T+NT	127T, 127NT	HCV	-	N.A
24	78	Female	Control	N	C1	Ovarian cancer	Liver metastasis	N.A
25	72	Male	Control	N	N.A	Leiomyosarcoma	Liver metastasis	N.A
26	80	Female	Control	N	N.A	Colon cancer	Liver metastasis	N.A
27	63	Male	Control	N	N.A	Colon cancer	Liver metastasis	N.A
28	79	Male	Control	N	C5	Colon cancer	Liver metastasis	N.A
29	38	Female	Control	N	N.A	Breast cancer	Liver metastasis	N.A
30	63	Female	Control	N	N.A	Colon cancer	Liver metastasis	N.A
31	63	Male	Control	N	C8	Colon cancer	Liver metastasis	N.A
32	59	Male	Control	N	N.A	Colon cancer	Liver metastasis	N.A
33	55	Female	Control	N	N.A	Pancreatic cancer	Liver metastasis	N.A

a Pair-Matched HCC with adjacent non tumour tissues. All NT were cirrhotic (F4).

b Edmondson-Steiner Grading system.

For patients affected by HCC, one biopsy from the tumour tissue and one from the adjacent non tumour liver parenchyma were taken, snap frozen in liquid nitrogen and kept at −80°C until RNA extraction. The histological examination of the adjacent non tumour liver parenchyma showed cirrhosis for all HCC patients. We refer to adjacent non tumour liver parenchyma as cirrhotic livers. For each biopsy, a mirror slide was available. Tumour cell content in HCC samples was greater than 90% and no sign of necrosis was observed in any biopsy. For patients operated for liver metastases, one biopsy of the liver parenchyma not affected by the metastasis was processed similarly. Liver parenchyma from patients operated for liver metastases was not affected by any chronic liver disease and pre-operative chemotherapy was usually stopped one month before surgery. The histological examination of these biopsies showed generally mild steatosis or fibrosis (F0-F1 based on Metavir score). We refer to these tissues as control livers.

Twenty samples from HCC patients (both tumour and matched adjacent cirrhotic tissues from 10 patients) and three control livers were selected and processed to undergo RNA-sequencing (Table 4). The rest of the samples underwent RNA extraction and RNA from all cases and controls were used for validation purposes.

Informed consent was obtained from each patient included in the study, and the study protocol conformed to the ethical guidelines of the 1975 Declaration of Helsinki.

### RNA extraction and library preparation

Total RNA extraction was performed with miRvana kit (Ambion). RNA quantity and quality were verified by Nanodrop (Thermo Scientific) and capillary electrophoresis (Bioanalyzer, Agilent) respectively. RNAs (3.5 μg) were rRNA depleted using either Ribominus (Life Techologies) or Ribozero kits (Epicentre). Barcoded libraries were prepared for each sample using SOLiD total RNA-Seq kit (Life Techologies) and following the manufacturer's instructions. Library profiles and concentration were verified with Bioanalyzer and Qubit (Life Technologies) before equimolar pooling of the libraries.

### RNA-sequencing

Paired-end sequencing was performed on a SOLiD platform (Life Techologies). Three cases (coded as 112, 122 and 127 tumours and matched-paired adjacent non tumour tissues) were sequenced using the SOLiD XL 5500 version, while the rest of the samples were sequenced using the updated SOLiD WildFire version.

Bioinformatics processing of raw data (.xsq file) was performed using LifeScope software (Life Technologies). The alignment of sequenced reads was performed on the human genome version hg19 GRCh37. Aligned reads were annotated using Gencode release 17, which includes 57,281 genes, of which 20,330 protein-coding genes, 13,333 long non-coding RNAs and 14,154 pseudogenes. A text table containing number of reads and FPKM (Fragment Per Kilobase of Exon per Million fragments mapped) value for each sample was generated and the data used for further analyses.

### RT-qPCR

One μg of total RNA was used to generate the cDNA, using the M-MLV reverse transcriptase (Life Technologies) and random hexamer primers, following the manufacturer's instruction. Quantitative real-time PCR (RT-qPCR) was performed in triplicate for each sample (20 HCC, 20 adjacent non tumour tissues and 10 control livers). The genes tested were *FAM99A*, *LINC01093*, *CRNDE*, *H19* and *HULC*. *SFRS4* was used as a reference gene, as it is reported to be very stable in liver disease context [50]. Moreover *SFRS4* is also expressed at similar levels in both tissue types in each of the pairs we analysed (data not shown). The primers used are reported in Table 5. The assays were performed using MESA GREEN qPCR MasterMix Plus (Eurogentec) and a CFX96 Real-Time PCR Detection System (Biorad). mRNA levels were calculated using the 2^−ΔCt^ method (ΔCt = ΔCt ^target gene^-ΔCt ^reference gene^).

**Table 5:** Primers used for RT-qPCR analysis on liver tissues

Gene	F-primer	R-primer
*LINC01093*	5-CCTTGTGACACTGAGATCAGCTA-3	5-ATCTCCCAGTCGGGTTTCCT-3
*CRNDE*	5-ACACGGCTTTCCGGAGTAGA-3	5-GCCAACATTTGGAGGAACCC-3
*FAM99A*	5-GTCCCTTGCCCTCTCTTGTC-3	5-ACACGCATCACAAAACAGCC-3
*H19*	5′-ATCGGTGCCTCAGCGTTC-3′	5′-AGAAACAGACCCGCTTCTTG-3′
*HULC*	5′-ACCTCCAGAACTGTGATCCAAAATG-3′	5′-TCTTGCTTGATGCTTTGGTCTG-3′
*SRFS4*	5′-GGCTACGGGAAGATCCTGGA-3′	5′-TGCATCACGCAGATCATCAA-3′

### Statistical analyses

Statistical analyses were carried out using R software version 3.1.3. Differential gene expression analysis was performed using the R package “edgeR” [51]. The package implements exact statistical methods for multigroup experiments. A particular feature of edgeR functionality is the empirical Bayes method that permits the estimation of gene-specific biological variation, even for experiments with minimal levels of biological replication [52]. We performed a filtering step excluding the genes whose expression was under the threshold of 1 count per million (1 cpm) in at least one sample. Read count normalization was performed using the standard function provided in edgeR package. Genes were considered as differentially expressed at FDR <0.05 and with absolute fold change higher than 2. The FDRs were calculated using the Benjamin-Hochberg procedure [53].

Correlation between the expression of a subset of differentially expressed long non-coding genes and their closest genes was performed as previously described using Pearson correlations [54].

Gene expression levels from RT-qPCR are expressed as mean (m) and standard deviation (sd). Means were compared in the different groups using the Wilcoxon test for paired tissues and the Kruskal-Wallis test when controls were compared with adjacent cirrhotic tissues or HCC tissues. χ^2^ test was used to compare categorical variables.

### Gene co-expression network analysis

The co-expression network construction was performed following the Weighted Gene Coexpression Network Analysis (WGCNA) approach previously described [55] using its R implementation [56]. We used WGCNA for analysing either differentially expressed genes or all genes expressed in at least one sample at a minimum level of 1 count per million reads. Read levels were normalized using the normalization factors provided by edgeR, as previously described [57].

## SUPPLEMENTARY FIGURE AND DATA












