# Supplementary material for: Identification of novel long non-coding RNAs deregulated in hepatocellular carcinoma using RNA-sequencing
Source: Oncotarget. 2016 Feb 13;7(22):31862–77. doi: 10.18632/oncotarget.7364 (PMC5077982; doi:10.18632/oncotarget.7364)
Supplement: Supplementary file 1 [file oncotarget-07-31862-s001.pdf]

Identification of novel long non-coding RNAs deregulated in hepatocellular carcinoma using RNA-sequencing

SUPPLEMENTARY FIGURE AND DATA

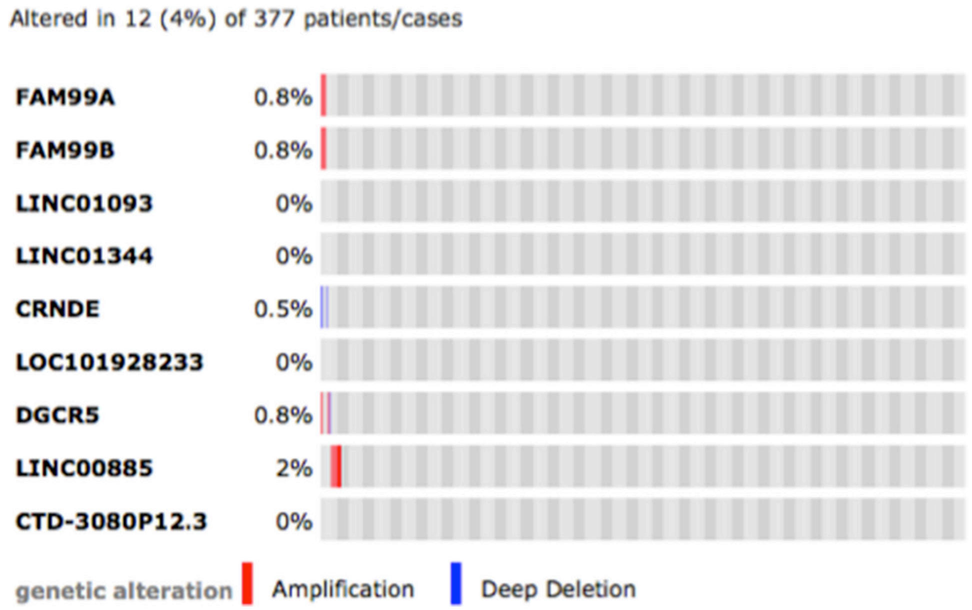

Supplementary Figure S1: Copy Number Alteration for eRNAs reported in HCC cases in the TCGA database, as reported by cBioportal (www.cbioportal.org).

Supplementary Document S1

See Supplementary File 1

Supplementary Document S2

See Supplementary File 2

Supplementary Document S3

See Supplementary File 3

Supplementary Document S4

See Supplementary File 4

Supplementary Document S5

See Supplementary File 5
